# LncRNA H19 knockdown promotes neuropathologic and functional recovery via the Nrf2/HO‐1 axis after traumatic brain injury

**DOI:** 10.1111/cns.14870

**Published:** 2024-07-25

**Authors:** Qiankang Chen, Biwu Wu, Ziyu Shi, Yana Wang, Yiwen Yuan, Xingdong Chen, Yuqing Wang, Jin Hu, Leilei Mao, Yanqin Gao, Gang Wu

**Affiliations:** ^1^ Department of Neurosurgery of Huashan Hospital, State Key Laboratory of Medical Neurobiology and MOE Frontiers Center for Brain Science Institutes of Brain Science, Fudan University Shanghai China

**Keywords:** long non‐coding RNA H19, neuroprotection, Nrf2, traumatic brain injury

## Abstract

**Aims:**

Traumatic brain injury (TBI) stands as a significant concern in public health, frequently leading to enduring neurological deficits. Long non‐coding RNA H19 (lncRNA H19) exerts a potential regulator role in the pathology of brain injury. This study investigates the effects of lncRNA H19 knockdown (H19‐KD) on the pathophysiology of TBI and its potential neuroprotective mechanisms.

**Methods:**

Controlled cortical impact was employed to establish a stable TBI mouse model. The expression levels of various genes in perilesional cortex and striatum tissue after TBI was detected by RT‐qPCR. AAV9‐shRNA‐H19 was injected into the lateral ventricle of mice to knockdown the expression of lncRNA H19. Various behavioral tests were performed to evaluate sensorimotor and cognitive functions after TBI. Immunofluorescence and Nissl staining were performed to assess brain tissue damage and neuroinflammation. The Nrf2 and HO‐1 expression was performed by Western blot.

**Results:**

After TBI, the expression of lncRNA H19 was elevated in perilesional tissue and gradually reverted to baseline. Behavioral tests demonstrated that H19‐KD significantly promoted the recovery of sensorimotor and cognitive functions after TBI. Besides, H19‐KD reduced brain tissue loss, preserved neuronal integrity, and ameliorated white matter damage at the histological level. In addition, H19‐KD restrained the pro‐inflammatory and facilitated anti‐inflammatory phenotypes of microglia/macrophages, attenuating the neuroinflammatory response after TBI. Furthermore, H19‐KD promoted activation of the Nrf2/HO‐1 axis after TBI, while suppression of Nrf2 partially abolished the neuroprotective effect.

**Conclusion:**

H19‐KD exerts neuroprotective effects after TBI in mice, partially mediated by the activation of the Nrf2/HO‐1 axis.

## Graphical abstract

Knockdown of the lncRNA H19 significantly promotes recovery from traumatic brain injury (TBI) in mice, as evidenced by reduced neuroinflammation, attenuated tissue damage including gray and white matter, and improved neurological function. This neuroprotective effect was significantly mediated by activation of the Nrf2/HO‐1 signaling pathway, revealing a potential target for therapeutic strategies for TBI.

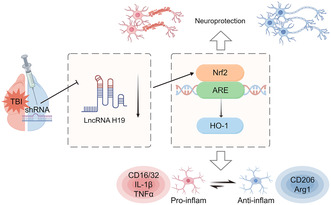

## 1 INTRODUCTION

TBI is a non‐congenital, non‐degenerative brain injury caused by direct or indirect mechanical forces, such as intense collisions, rapid acceleration‐deceleration, and rotational brain movements. TBI has the highest incidence among all common neurological disorders, affecting over 50 million people worldwide annually, and stands as a leading cause of death in the young adult population.^1^ Survivors of TBI often experience a range of cognitive, sensory, and motor impairments, greatly diminishing their quality of life and placing considerable burdens on families and societies.^2^, ^3^ Despite ongoing advancements in clinical treatments for TBI spanning several decades, enhancing the prognosis for TBI patients remains a formidable challenge.^4^

The pathophysiological mechanisms of TBI are intricate, comprising both the primary mechanical disruption of brain tissue and secondary diffuse brain injury.^5^, ^6^ The primary injuries occur immediately upon the injury event and are irreversible, including brain concussion, contusions, and intracranial hematomas. Subsequently, secondary injuries develop over hours to years, characterized by processes such as neuroinflammation, oxidative stress, cell apoptosis, and autophagy.^7^, ^8^ At the histological level, damage or loss of both gray and white matter caused by the cascade of these injury processes impairs neurological functions in patients. Reduction in gray matter volume of TBI patients is associated with cognitive, emotional, and motor control impairments.^9^, ^10^, ^11^ White matter damage is also a crucial aspect of TBI, widely considered as a major contributor to TBI‐related impairments in higher brain functions.^12^, ^13^ Hence, it is imperative to identify targets for enhancing recovery from secondary gray and white matter damage, thereby promoting neurofunctional rehabilitation in TBI.

Long non‐coding RNAs (lncRNAs) were initially overlooked due to their lack of protein‐coding biological functions.^14^ The regulation of lncRNA in gene transcription involves various mechanisms closely associated with physiological and pathological processes, including cell differentiation, tumorigenesis, and inflammation.^15^, ^16^, ^17^ LncRNA H19, one of the earliest identified imprinting lncRNA, has a length of 2.3 kb and exhibits high evolutionary conservation.^18^ In central nervous system (CNS) injuries such as cerebral hemorrhage and cerebral ischemia/hypoxia, lncRNA H19 regulates inflammatory responses, oxidative stress, cell apoptosis, and other pathological processes through multiple pathways.^19^, ^20^ LncRNA H19 is also engaged in maintaining the integrity of gray and white matter following brain injury.^21^

Microglia, resident immune cells in the brain, migrate to the injury site and trigger a series of inflammatory reactions that disrupt the blood–brain barrier and cause neuronal loss following TBI.^22^, ^23^, ^24^ Pro‐inflammatory microglia, once activated and polarized, release numerous pro‐inflammatory cytokines, intensifying the inflammatory response and exacerbating tissue damage. Conversely, anti‐inflammatory microglia promote brain recovery by clearing cellular debris, alleviating local inflammation, and releasing trophic factors.^25^, ^26^ Microglia‐mediated inflammatory responses play a crucial role in brain damage following TBI, particularly in white matter injury.^27^ Therefore, targeting the regulation of microglial activation may offer an effective strategy for inflammation‐mediated TBI treatment interventions. Increasing evidence suggests that various lncRNA, including lncRNA H19, can participate in regulating neuroinflammatory processes by modulating microglial activation.^28^, ^29^, ^30^

Nuclear factor erythroid 2‐related factor 2 (Nrf2), a transcription factor that belongs to the basic leucine zipper protein family, plays a crucial role in cellular defense against oxidative stress in the central nervous system (CNS). Nrf2 exerts a protective effect during oxidative stress via binding to antioxidant response elements of endogenous protective genes.^31^, ^32^, ^33^ Numerous studies have indicated that Nrf2 serves as a key endogenous factor in defending against oxidative stress in the brain. Furthermore, it plays a neuroprotective role in TBI by modulating microglial function and influencing neuroinflammation.^34^, ^35^, ^36^ Heme oxygenase‐1 (HO‐1), an inducible enzyme responsible for heme degradation, acts as an important downstream antioxidant in the Nrf2 pathway, a series of in vivo and in vitro experiments highlighting its critical role in the inflammatory process.^37^, ^38^, ^39^ Current research has suggested that in myocardial injury, H19 can modulate the Nrf2/HO‐1 axis to some extent.^40^ Specifically, H19 knockdown (H19‐KD) upregulates the Nrf2 and HO‐1 expression, thereby alleviating oxidative stress and inflammatory responses.^41^

Previous studies have revealed that downregulating the expression of H19 can enhance the midline crossing sprouting of intact corticospinal tract axons in the spinal cord mediated by IGF1 and the mTOR signaling pathway, thereby improving sensory‐motor function in middle cerebral artery occlusion (MCAO) rats.^42^ This suggests the significant potential value of H19 in protecting against CNS injury. Clinical observations, supported by multimodal monitoring, have unveiled that the progression of TBI often involves manifestations such as cerebral hemorrhage, cerebral ischemia/hypoxia, and other pathological processes. This strongly implies that alternations in the expression of H19 may occur after TBI. However, currently, there is no report on the study of H19 in TBI. Further research is warranted to investigate whether regulating its expression can modulate the polarization state of microglia after TBI, thereby influencing neuroinflammation, reducing damage to gray and white matter, and promoting neurological function recovery. Moreover, it is important to determine whether this effect is mediated by the involvement of H19 in regulating the Nrf2/HO‐1 axis.

In this study, we achieved the knockdown of H19 in the brain using the adeno‐associated virus vector AAV9‐shRNA‐H19. Our findings underscore the critical role of H19‐KD in regulating microglial polarization, ameliorating neuroinflammation, alleviating gray and white matter damage in the brain, and promoting neurological function recovery after TBI. Furthermore, we molecularly confirmed that H19‐KD exerts its neuroprotective effect by promoting the activation of the Nrf2/HO‐1 axis.

## 2 METHODS

### 2.1 Animals and experimental design

The C57BL/6 mice used in this study were purchased from Shanghai Jihui Laboratory Animal Care Co., Ltd. Mice were housed in individually ventilated cages (IVC) under pathogen‐free conditions, with suitable and stable environmental temperature and humidity. A 12 h light–dark cycle was maintained, and the mice were provided ad libitum access to food and water. Before any experiments, the mice were given at least 3 days to acclimate to the surroundings. All animal experiments in this study were approved by the Animal Ethics Committee of the Department of Laboratory Animal Science, Fudan University (Approval number 2021JS‐329).

Adult male mice (aged 8–10 weeks, weight 23 – 27 g) were selected as experimental subjects. The mice were randomly assigned to different groups, which included a wild‐type (WT) sham‐operated group (Veh‐sham), an H19‐KD sham‐operated group (KD‐sham), a WT surgery group (Veh‐TBI), an H19‐KD surgery group (KD‐TBI), and the Nrf2 inhibitor group (KD‐Sham+ML, KD‐TBI + ML). These groups underwent AAV injection, TBI modeling, and inhibitor injection, respectively, for subsequent research purposes.

### 2.2 AAV injection

The AAV‐shRNA‐H19 and negative control virus solutions used in this study were obtained from Shanghai Genechem Co., Ltd. Fourteen days before TBI, AAV‐shRNA‐H19 or the negative control virus solution was injected into the right lateral ventricle of the mice by stereotactic localization (Data S1).

### 2.3 TBI model in mice

The TBI mouse model was induced using an established controlled cortical impact (CCI).^43^ This model does not cause postoperative death in mice under normal conditions. Mice were anesthetized and fixed as described, and the skull was exposed. The center of controlled cortical impact (CCI) was identified 2 mm lateral to the midline and 0.5 mm posterior to the bregma. A craniotomy was performed consistently on the right side using a skull drill with a cranial window approximately 4 mm in diameter. A cortical impactor device (TBI 0310, Precision Systems and Instrumentation) equipped with a flat‐tip impactor head with a diameter of 3 mm was used to impact the brain tissue, according to the set parameters: velocity of 3.5 m/s, depth of 1.5 mm, and duration of 150 ms. After CCI, the scalp was carefully sutured and disinfected, and the mice were placed on a heating pad to maintain their body temperature until they fully recovered from anesthesia. Sham‐operated mice underwent all surgical procedures except for the CCI.

### 2.4 Neurofunctional behavioral tests

The neurobehavioral deficits and recovery following TBI were evaluated using a range of behavioral experiments (Data S1). Sensory motor function tests included foot fault, rotarod, adhesive removal, and wire hanging test. Cognitive memory function was assessed using novel object recognition and a three‐chamber social test. All assessments were conducted using blind methods to ensure unbiased analysis.

### 2.5 Immunofluorescence and Cresyl violet Nissl staining

Brain sections were obtained and subjected to immunofluorescence and cresyl violet Nissl staining (Data S1).

### 2.6 Real‐time qPCR

Fresh perilesional tissue of the mouse brain was taken and RNA was extracted for RT‐qPCR (Data S1).

### 2.7 Western blotting

Fresh perilesional tissue of the mouse brain was similarly taken and total protein was extracted for Western blot (Data S1).

## 3 RESULTS

### 3.1 LncRNA H19 knockdown promotes recovery of sensorimotor and cognitive memory functions after TBI in mice

**FIGURE 1:**
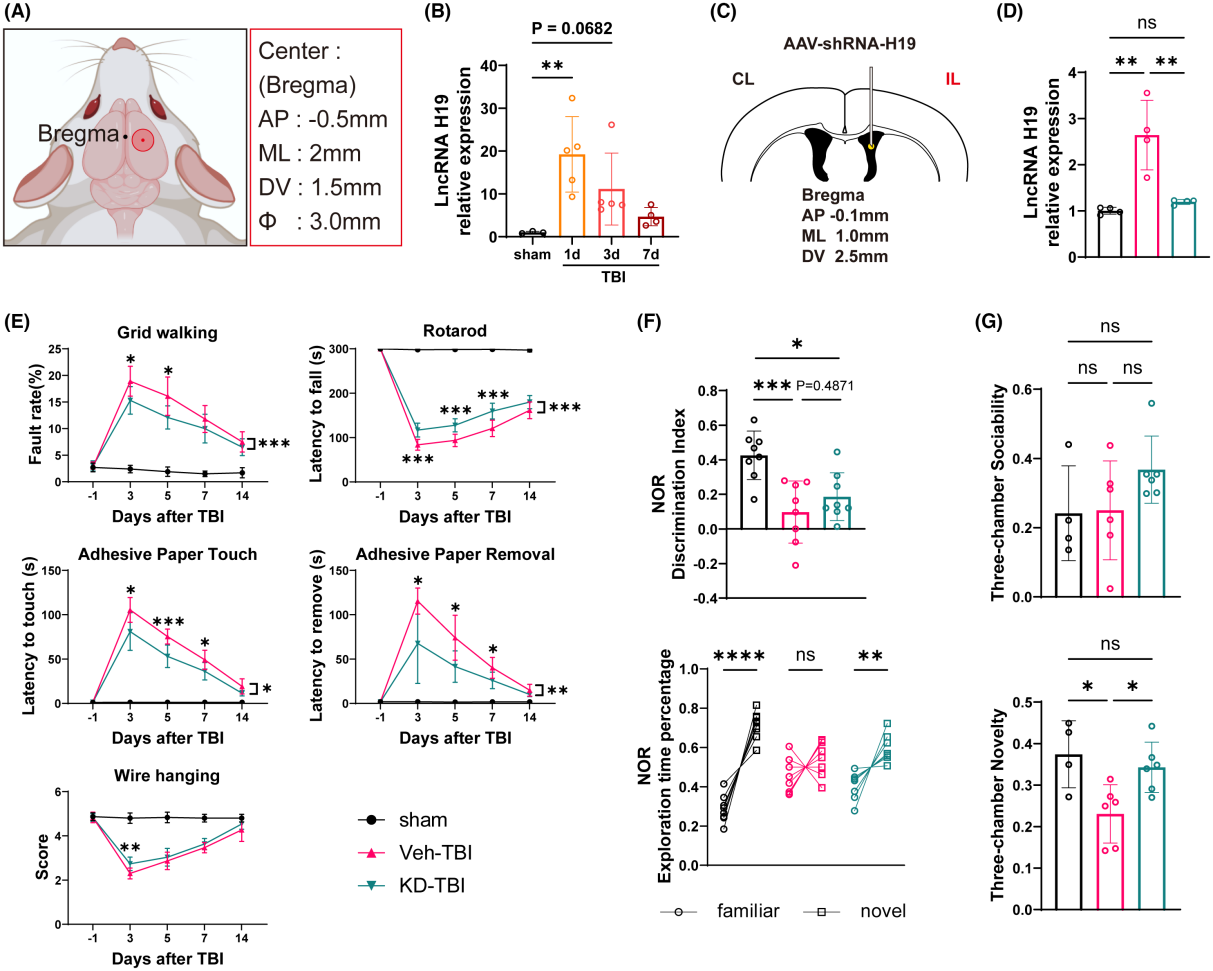
LncRNA H19 knockdown promotes recovery of sensorimotor and cognitive memory functions after TBI in mice. (A) Schematic representation of CCI surgery. (B) Quantification of H19 mRNA expression after TBI. (C) Schematic illustration of stereotactic injection into the lateral ventricle. (D) Quantification of H19 m RNA expression in Sham, Veh‐TBI, and KD‐TBI groups (*n* = 4–5/group). (E) Results of sensorimotor behavioral test (*n* = 10/group). (F) Discrimination index and Exploration Time Percentage of novel object recognition test (*n* = 8/group). (G) Sociability and novelty preferences assessed by the three‐chamber social test (*n* = 4–6/group). Data are presented as mean ± SD. Statistical analyses were performed by one‐way or two‐way ANOVA and Tukey's multiple comparisons test. *p < 0.05, **p < 0.01, ***p < 0.001, ns: No significance, KD‐TBI vs. Veh‐TBI, or as indicated.

To investigate the involvement of H19 in the physiological and pathological processes after TBI, we first established a stable mouse TBI model through CCI surgery (Figure 1A). Expression levels of H19 in the perilesional cortex (CTX) and striatum (STR) tissues of WT mice were assessed at 1, 3, and 7 days after sham surgery or TBI using RT‐qPCR. Results showed upregulated expression of H19 in the perilesional tissues post‐TBI compared to the sham group, peaking at day 1 and showing a trend of upregulation at day 3 (Figure 1B). This aligns with expectations, suggesting H19 as a stress‐induced product during the acute phase of TBI pathology. To examine how manipulating H19 expression affects brain injury and neurological function recovery after TBI, we constructed an adeno‐associated virus vector AAV9‐shRNA‐H19, injected into the right lateral ventricle of mice 14 days before TBI, ensuring ample time for infection and shRNA expression (Figure 1C). RT‐qPCR at 7 days post‐TBI showed that the Veh‐TBI group maintained higher H19 expression in the perilesional tissues compared to the sham group, while the H19 expression in the KD‐TBI group did not significantly differ from sham (Figure 1D). This indicates effective suppression of H19 upregulation in the perilesional tissues after TBI by intracerebroventricular AAV9‐shRNA‐H19 injection, restoring levels to normal and achieving successful H19‐KD in the mouse brain.

To explore the neuroprotective effects of H19‐KD and its impact on neurological dysfunction after TBI, we conducted comprehensive behavioral tests. To exclude the influence of H19 inhibition on the physiological state of mice, we categorized animals into the control sham surgery group (Veh‐sham), H19‐KD sham surgery group (KD‐sham), control injury group (Veh‐TBI), and H19‐KD injury group (KD‐TBI). In sensorimotor behavioral tests, no significant difference was observed between the Veh and KD groups under sham conditions, indicating that H19‐KD did not affect mouse neurological function under physiological conditions (Figure S1A–D). Compared to the sham group, mice in the Veh‐TBI group showed severe sensory‐motor dysfunction from days 3 to 14 post‐TBI in the grid walking test, rotarod test, adhesive remove test, and wire hanging test (Figure 1E). In contrast, H19‐KD significantly reduced the forelimb fault rate in the grid walking test, implying improved limb sensation and coordination. In the rotarod test, KD‐TBI mice exhibited longer retention times on the rod, indicating enhanced endurance abilities of exercise. They also showed significantly reduced times in perceiving and removing the adhesive tape in the adhesive remove test, suggesting better limb sensation and motor skills. Additionally, KD‐TBI mice exhibited improved muscle strength in the wire hanging test at 3 days post‐injury. In conclusion, these behavioral test results suggest that H19‐KD contributes to the recovery of sensorimotor function in mice after TBI (Figure 1E).

Similarly, to explore whether H19‐KD promotes cognitive memory recovery in mice after TBI, we conducted novel object recognition and three‐chamber social tests on mice. In the novel object recognition test, a higher Exploration Time Percentage and Discrimination Index indicate a better object recognition ability. Compared to the sham group, mice in the Veh‐TBI group showed reduced the ability to distinguish between new and old objects, while KD‐TBI mice exhibited a trend toward recovery (Figure 1F). The three‐chamber social test indicated that TBI did not affect the social abilities of mice, while KD‐TBI group mice exhibited a more proactive social trend. Additionally, compared to the sham group mice, Veh‐TBI mice showed a significant decrease in social novelty preference, while KD‐TBI mice showed enhanced social novelty preference, almost returning to normal levels (Figure 1G), which showed that KD‐TBI group mice had a more pronounced cognitive ability and social preference for unfamiliar objects. Together, these results suggest that H19‐KD promotes cognitive memory recovery to some extent in mice after TBI, indicating its neuroprotective effect after TBI.

### 3.2 LncRNA H19 knockdown reduces brain tissue loss and white matter damage following TBI in mice

**FIGURE 2:**
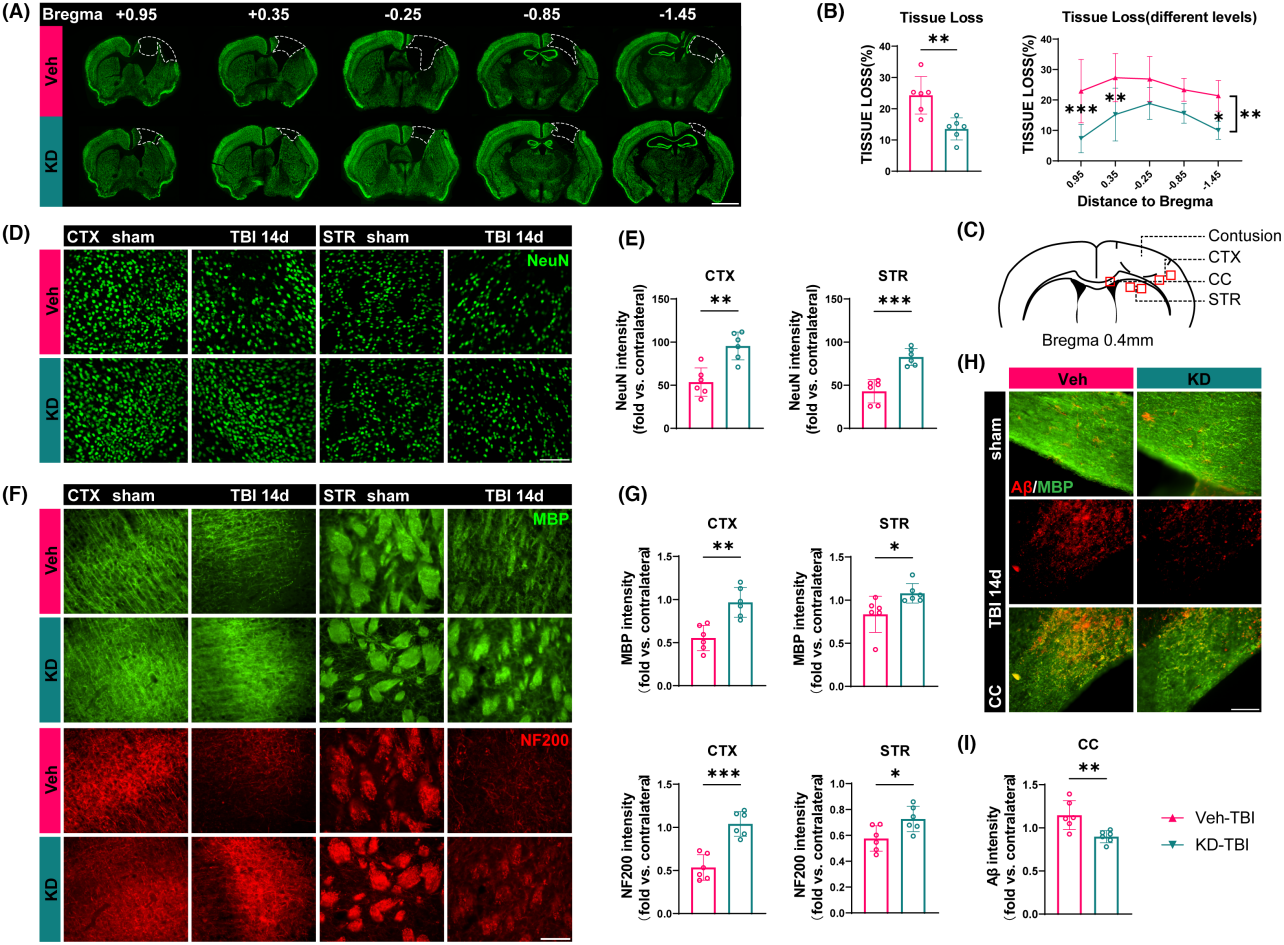
LncRNA H19 knockdown reduces brain tissue loss and white matter damage after TBI in mice. (A) Representative images of NeuN immunostaining (scale bar = 2 mm). (B) Quantification of total brain tissue loss volume or brain tissue loss area from consecutive coronal sections starting at +0.95 mm from the bregma. (C) Schematic representation of perilesional brain regions for immunostaining image capture (D) Representative images of NeuN immunostaining in the perilesional CTX and STR regions. (E) Quantification of NeuN‐positive cells in the perilesional CTX and STR regions. (F) Representative images of MBP and NF200 immunostaining in the perilesional CTX and STR regions. (G) Quantification of MBP and NF200 fluorescence intensity in the perilesional CTX and STR regions. (H) Representative images of MBP and Aβ co‐staining in the perilesional CC region (scale bar = 100 μm) (I) Quantification of Aβ fluorescence intensity in the perilesional CC region. Data are presented as mean ± SD, *n* = 6/group. Statistical analyses were performed by Unpaired *t*‐test or two‐way ANOVA and Tukey's multiple comparisons test. *p < 0.05, **p < 0.01, ***p < 0.001, KD‐TBI vs. Veh‐TBI, or as indicated.

Histological damage severity after TBI is a crucial indicator in clinical assessment. We utilized immunofluorescence staining of the neuronal nuclear marker NeuN to evaluate brain tissue damage in mice 14 days after TBI (Figure 2A). Results revealed smaller overall brain damage and reduced damage area at different levels in the KD group compared to the Veh group, indicating a protective effect of H19‐KD against brain tissue damage after TBI (Figure 2B). Since neurons are the essential constituents of gray matter structures, we further analyzed neuronal loss in the perilesional CTX and STR regions 14 days after TBI (Figure 2D). Compared to the sham group, Veh‐TBI mice exhibited significant reduction in NeuN‐positive neurons in CTX and STR, while KD‐TBI mice demonstrated more NeuN‐positive neurons in these regions (Figure 2E), suggesting that H19‐KD significantly reduced brain tissue defects and neuronal loss in mice after TBI, thereby exerting a protective effect against secondary gray matter injury.

White matter, composed of myelinated axonal fibers, is a critical factor in the pathological process of TBI, leading to long‐term sensorimotor and cognitive impairments.^13^ To investigate the protective effect of H19‐KD on white matter integrity, we utilized myelin basic protein (MBP) and neurofilaments (NF200) as markers for myelin sheaths and axonal fibers, respectively. Immunofluorescence staining was used to detect white matter integrity in the perilesional CTX and STR of the mouse brain 14 days after TBI (Figure 2F). Results indicated that compared to the sham group, the fluorescence intensity of MBP and NF200 in the perilesional CTX and STR regions was decreased in the Veh‐TBI group, indicating significant myelin protein loss and axonal damage following TBI. Conversely, compared to the Veh‐TBI group, the KD‐TBI group exhibited significantly increased fluorescence intensity of MBP and NF200 in the perilesional CTX and STR regions (Figure 2G), suggesting that H19‐KD effectively preserves white matter structure integrity by alleviating myelin loss and axonal damage induced by TBI.

Primary axonal injury caused by TBI can lead to axonal degeneration and impair axoplasmic transport. Subsequent deposition of β‐amyloid protein (Aβ) may induce secondary neuronal death and white matter damage, resulting in cognitive impairment and triggering neurodegenerative diseases such as Alzheimer's disease.^44^ Reducing the aggregation of these abnormal proteins to mitigate secondary tissue damage is crucial for promoting neurological recovery after TBI.^45^ Therefore, we conducted immunofluorescence co‐staining for MBP and Aβ in the corpus callosum (CC), an axon‐rich region of the mouse brain, 14 days after TBI to observe Aβ accumulation (Figure 2H). Results showed a significantly enhanced Aβ fluorescence signal in the perilesional CC region of the Veh‐TBI group compared to the sham group. In contrast, the fluorescence intensity of Aβ in the perilesional CC region of the KD‐TBI group was significantly reduced compared to the Veh‐TBI group, indicating improved Aβ deposition and aggregation (Figure 2I). This finding suggests that H19‐KD may reduce the aggregation of Aβ induced by axonal injury after TBI, thereby offering protection against white matter damage.

### 3.3 LncRNA H19 knockdown ameliorates the inflammatory response by modulating the microglia/macrophages polarization after TBI in mice

**FIGURE 3:**
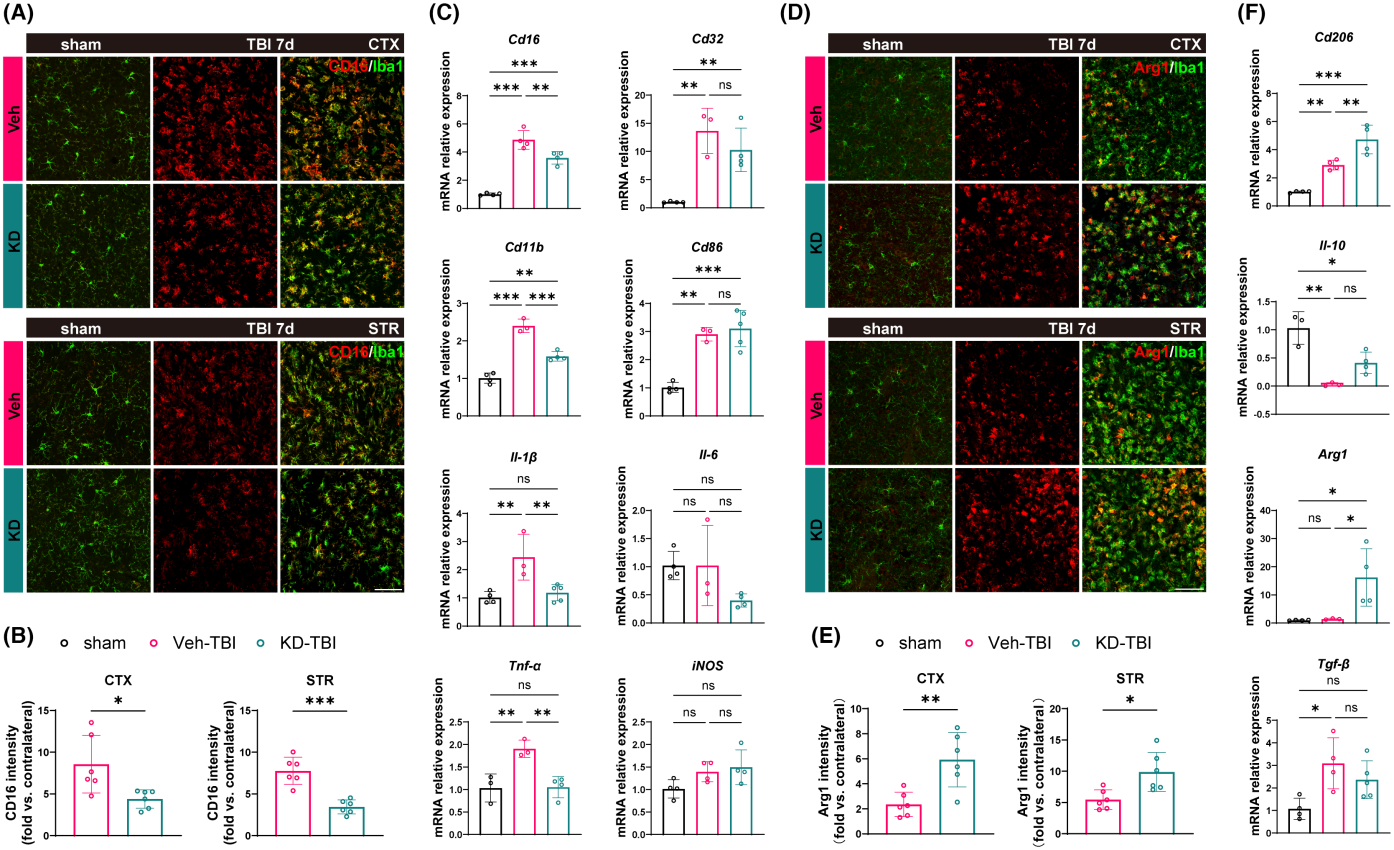
LncRNA H19 knockdown ameliorates the inflammatory response by modulating the polarization state of microglia/macrophages after TBI in mice. (A) Representative images of CD16 and Iba1 co‐staining in the perilesional CTX and STR regions 7 days after TBI (scale bar = 100 μm). (B) Quantification of CD16 fluorescence intensity in the perilesional CTX and STR regions (*n* = 6/group). (C) mRNA expression levels of pro‐inflammatory cytokines and markers by RT‐qPCR 7 days after TBI (*n* = 3–4/group). (D) Representative images of Arg1 and Iba1 co‐staining in the perilesional CTX and STR regions 7 days after TBI (scale bar = 100 μm). (E) Quantification of Arg1 fluorescence intensity in the perilesional CTX and STR regions (*n* = 6/group). (F) mRNA expression levels of anti‐inflammatory cytokines and markers analyzed by RT‐qPCR 7 days after TBI (*n* = 3–4/group). Data are presented as mean ± SD. Statistical analyses were performed by Unpaired t‐test or one‐way ANOVA and Tukey's multiple comparisons test. *p < 0.05, **p < 0.01, ***p < 0.001, ns: No significance, as indicated.

TBI induces activation of the central immune system, represented by microglia, leading to a robust neuroinflammatory response pivotal in secondary injury processes.^23^ To explore whether H19‐KD can alter microglia polarization to influence neuroinflammation, we first performed immunofluorescence co‐staining using the ionized calcium‐binding adapter molecule 1 (Iba1, a microglia/macrophage marker) and CD16 (a pro‐inflammatory marker) to assess the activation status of pro‐inflammatory (Iba1^+^/CD16^+^) microglia/macrophages in the perilesional CTX and STR regions of the mouse brain 7 days after TBI (Figure 3A). Compared to the sham group, pro‐inflammatory microglia/macrophages in the perilesional CTX and STR regions of the Veh‐TBI group were significantly activated. Importantly, compared to the Veh‐TBI group, the fluorescence intensity of Iba1^+^/CD16^+^ in the perilesional areas markedly decreased in the KD‐TBI group (Figure 3B), indicating that H19‐KD suppressed pro‐inflammatory microglia/macrophages activation in the perilesional area after TBI. Additionally, we quantitatively analyzed mRNA expression of various pro‐inflammatory markers in the perilesional brain tissue of mice 7 days after TBI using RT‐qPCR. Results showed that compared to the sham group, the transcriptional expression levels of some pro‐inflammatory markers (CD16/32, CD11b, CD86) and pro‐inflammatory cytokines (IL‐1β, TNF‐α) were significantly increased in the brains of the Veh‐TBI group, indicating a severe inflammatory response induced by TBI in brain tissue. In contrast, the expression of some pro‐inflammatory markers (CD16/32, CD86) and pro‐inflammatory cytokines (IL‐1β, TNF‐α) significantly decreased in the brains of the KD‐TBI group compared to the Veh‐TBI group (Figure 3C). This suggests that H19‐KD can reduce neuroinflammation by inhibiting pro‐inflammatory microglia/macrophage activation.

Similarly, we performed co‐staining of arginase 1 (Arg1, an anti‐inflammatory marker) and Iba1 to observe the activation status of anti‐inflammatory (Iba1+/Arg1+) microglia/macrophages in the perilesional CTX and STR regions of the mouse brain 7 days after TBI (Figure 3D). Compared to the sham group, some anti‐inflammatory microglia/macrophages in the perilesional CTX and STR regions of the Veh‐TBI group showed a tendency toward activation. Interestingly, the fluorescence intensity of Iba1+/Arg1+ in the perilesional region significantly increased in the KD‐TBI group compared to the Veh‐TBI group (Figure 3E), indicating that H19‐KD promoted the activation of anti‐inflammatory microglia/macrophages in the perilesional region after TBI. RT‐qPCR results also showed that although anti‐inflammatory microglia/macrophages were activated in the perilesional tissue of the Veh‐TBI group, the transcriptional expression levels of Arg1 did not significantly increase, and the transcriptional expression levels of the anti‐inflammatory cytokine IL‐10 were significantly inhibited. Conversely, the transcriptional expression levels of the anti‐inflammatory markers CD206 and Arg1 significantly upregulated in the brains of the KD‐TBI group, with a trend toward recovery of IL‐10 expression, albeit without statistical difference between the two groups (Figure 3F). This indicates that H19‐KD improved the neuroinflammatory status in the brain after TBI by promoting the activation of anti‐inflammatory microglia/macrophages. Notably, there were no significant differences between the Veh and KD groups under sham conditions in all the experiments. Overall, these results suggest that H19‐KD can ameliorate the neuroinflammatory milieu and alleviate neuroinflammation by regulating the inflammatory polarization state of microglia/macrophages after TBI, thus exerting a neuroprotective effect.

### 3.4 LncRNA H19 knockdown promotes the activation of the Nrf2/HO‐1 axis after TBI in mice

**FIGURE 4:**
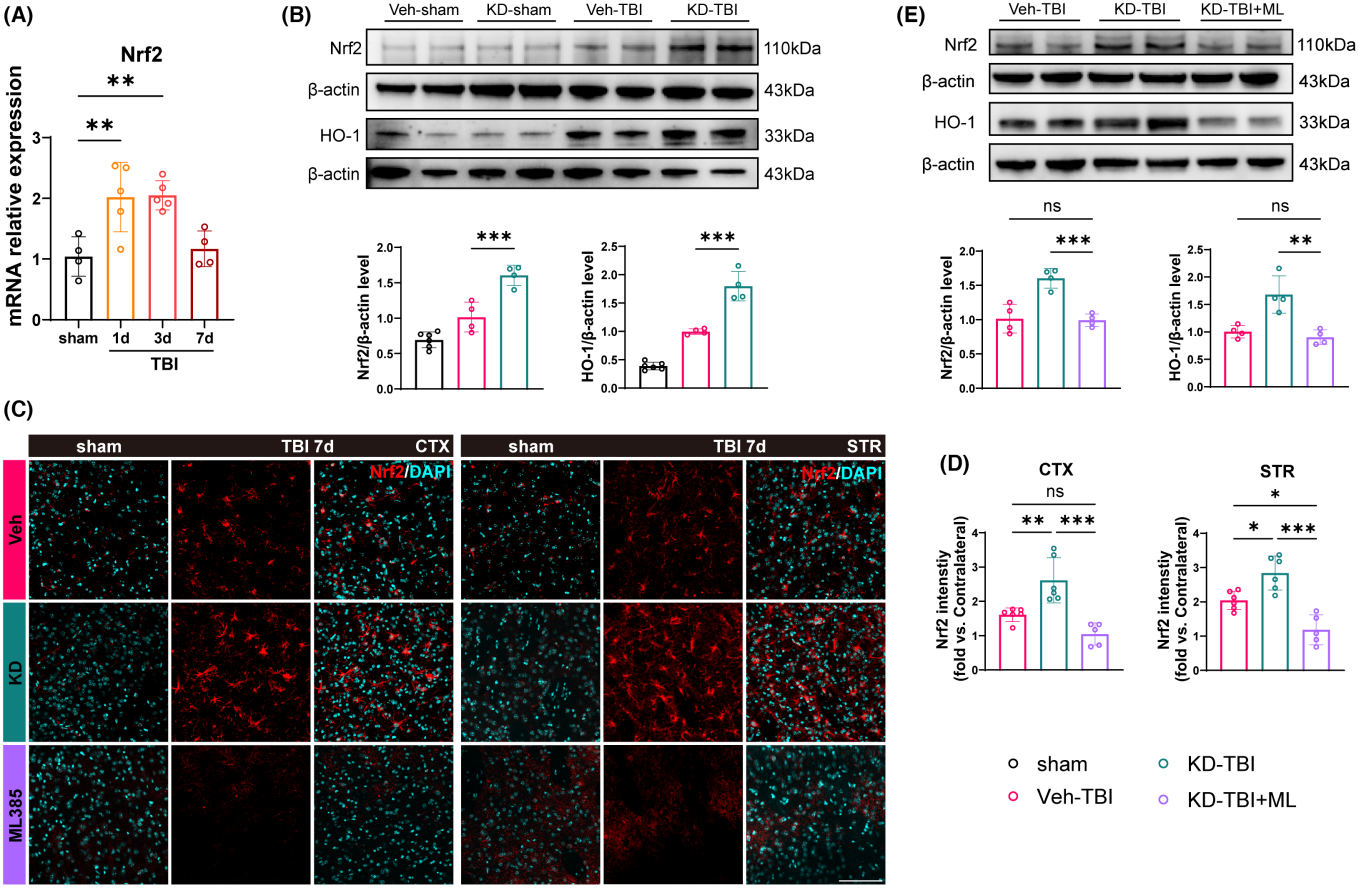
LncRNA H19 knockdown eliminates its inhibition of Nrf2/HO‐1 axis activation after TBI in mice. (A) Quantification of *Nrf2* mRNA expression levels after TBI in WT mice (*n* = 4–5/group). (B) Representative images and quantification of Western blotting for Nrf2 and HO‐1 protein expression 7 days after TBI. (C) Representative images of Nrf2 and DAPI immunostaining in the perilesional CTX and STR regions (scale bar = 100 μm). (D) Quantification of Nrf2 fluorescence intensity in the perilesional CTX and STR regions 7 days after TBI (*n* = 5–6/group). (E) Representative images and quantification of Western blotting for Nrf2 and HO‐1 protein levels in the perilesional area 7 days after TBI (*n* = 4/group). Data are presented as mean ± SD. Statistical analyses were performed by one‐way ANOVA and Tukey's multiple comparisons test. *p < 0.05, **p < 0.01, ***p < 0.001, ns: No significance, as indicated.

Previous studies have found that H19 participates in the regulation of the Nrf2/HO‐1 axis in various diseases.^32^, ^35^ To investigate its potential role in TBI, we first assessed Nrf2 expression levels in perilesional tissues of WT mice on day 1, 3, and 7 after sham surgery and TBI using RT‐qPCR. The results revealed a apparent upregulation of Nrf2 mRNA expression on day 1 and 3 post‐TBI compared to the sham group, with levels returning close to baseline by day 7 (Figure 4A). This suggests a transient increase in Nrf2 expression in the perilesional tissues after TBI. Subsequently, we examined whether H19‐KD could influence the Nrf2/HO‐1 axis post‐TBI using western blot to detect the protein expression of Nrf2 and its downstream target HO‐1 in the perilesional tissues on day 7 post‐TBI. Under sham conditions, there was no significant difference in Nrf2 and HO‐1 expression between Veh and KD mice, suggesting that H19‐KD did not affect the expression of Nrf2 and HO‐1 under physiological conditions (Figure S2). However, statistical analysis showed an upward trend in Nrf2 and HO‐1 protein levels in the Veh‐TBI group compared to the sham group, indicating the activation of Nrf2/HO‐1 axis in the perilesional tissues post‐TBI. Interestingly, the KD‐TBI group exhibited significantly increased protein levels of Nrf2 and HO‐1 compared to the Veh‐TBI group (Figure 4B), indicating that H19‐KD further promoted the activation of the Nrf2/HO‐1 axis after TBI.

To further clarify whether the activation of the Nrf2/HO‐1 axis is a downstream target of H19‐KD participating in its neuroprotective effect on TBI mice, we randomly divided TBI mice into three groups: Veh‐TBI group, KD‐TBI group, and KD‐TBI+ML group (treated with the Nrf2 inhibitor ML385). First, we performed Nrf2 immunofluorescence staining to detect the expression of Nrf2 in the perilesional CTX and STR of mice from each group on day 7 after TBI (Figure 4C). Results showed that the fluorescence intensity of Nrf2 in the perilesional CTX and STR of the KD‐TBI group mice was significantly higher than that in the Veh‐TBI group, further demonstrating that H19‐KD could promote the Nrf2 expression in the perilesional tissues after TBI. Notably, the fluorescence intensity of Nrf2 in the KD‐TBI+ML group was significantly decreased compared to the KD‐TBI group, even significantly lower than that in the Veh‐TBI group in the STR region, suggesting that ML385 treatment could completely abolish Nrf2 upregulation induced by H19‐KD after TBI (Figure 4D). Subsequently, western blot analysis of Nrf2 and HO‐1 protein levels in the perilesional tissue from each group showed significantly decreased levels in the KD‐TBI+ML group compared to the KD‐TBI group, with no significant difference compared to the Veh‐TBI group (Figure 4E). These findings, consistent with immunofluorescence results, affirm that ML385 treatment abolished Nrf2/HO‐1 axis activation induced by H19‐KD after TBI. Taken together, these results indicate that H19‐KD regulates the downstream Nrf2/HO‐1 axis after TBI and suggest that this pathway may serve as a target for the neuroprotective role of H19‐KD.

### 3.5 The neuroprotective effect of lncRNA H19 knockdown after TBI is mediated by activation of the Nrf2/HO‐1 axis

**FIGURE 5:**
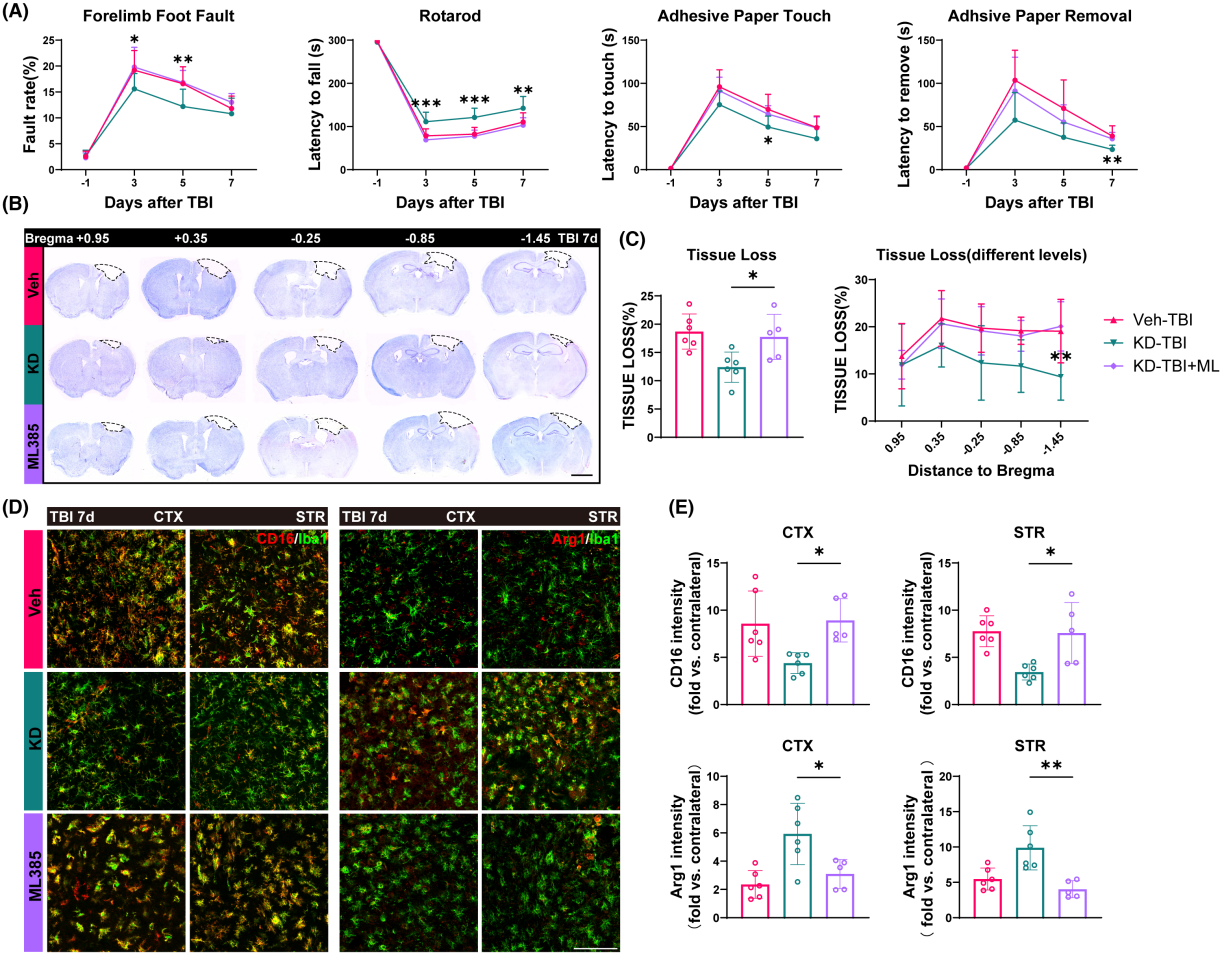
Inhibition of the Nrf2/HO‐1 axis partially abolishes the neuroprotective effect of lncRNA H19 knockdown after TBI. (A). Results of behavior tests. (B) Representative images of CV staining (scale bar = 2 mm). (C) Quantification of brain tissue loss. (D) Representative images of CD16/Arg1 and Iba1 co‐staining in the perilesional CTX and STR regions (scale bar = 100 μm). (E) Quantification of CD16/Arg1 fluorescence intensity in the perilesional CTX and STR regions of Veh, KD, and ML385‐treated mice 7d after TBI (*n* = 6/group). Data are presented as mean ± SD. *n* = 10/group. Statistical analyses were performed by one‐way/two‐way ANOVA and Tukey's multiple comparisons test. *p < 0.05, **p < 0.01, ***p < 0.001, KD‐TBI + ML vs. KD‐TBI, or as indicated.

Next, we further validated whether the neuroprotective effect of H19‐KD is mediated by the activation of the Nrf2/HO‐1 axis through a series of behavioral and histological tests. Using ML385 to inhibit the Nrf2/HO‐1 axis, we randomly divided TBI mice into the Veh‐TBI group, KD‐TBI group, and KD‐TBI + ML group as we did previously. Also, to determine whether ML385 injection would affect mice in normal physiological state, we added the KD‐sham+ML group compared with the KD‐sham+DMSO group. The results showed that ML385 injection does not lead to a worse outcome in mice in normal physiological states (Figure S3). In the grid walking test, the KD‐TBI + ML group mice exhibited significantly higher forelimb fault rates on 3 and 5 days after injury compared to the KD‐TBI group. In the rotarod test, the KD‐TBI + ML group mice showed significantly shorter duration of stay on the rotating rod from day 3 to 7 after injury compared to the KD‐TBI group, approaching the levels of the Veh‐TBI group. Moreover, in the adhesive removal test, the KD‐TBI + ML group mice spent significantly prolonged time perceiving and removing the adhesive tape 5 and 7 days after injury compared to the KD‐TBI group (Figure 5A). These results collectively indicate that the inhibition of the Nrf2/HO‐1 axis by ML385 partially reverses the beneficial effects of H19‐KD on sensorimotor neurofunction in TBI mice.

Subsequently, we conducted Nissl staining using Cresyl Violet (CV) on brain tissue sections from mice 7 days post‐TBI at different levels to assess brain tissue damage (Figure 5B). Analysis revealed that, compared to the KD‐TBI group, mice in the KD‐TBI + ML group exhibited a significantly increased overall extent of brain tissue damage, approaching the level observed in the Veh‐TBI group. Additionally, comparative analysis at multiple levels revealed an increasing trend in brain tissue damage area in each level of the KD‐TBI + ML group compared to the KD‐TBI group, particularly noticeable in levels closer to the hippocampal region (Figure 5C). This result indicates that the inhibition of the Nrf2/HO‐1 axis activation eliminates the protective effect of H19‐KD on secondary brain tissue damage in mice after TBI.

Finally, we performed immunofluorescence co‐staining of Iba1 with the pro‐inflammatory marker CD16 and the anti‐inflammatory marker Arg1 to observe the polarization states of microglia/macrophages in the perilesional CTX and STR of mice 7 days after TBI (Figure 5D). The fluorescence intensity of CD16 in the peri‐injury CTX and STR of the KD‐TBI + ML group was significantly increased compared to the KD‐TBI group, while the fluorescence intensity of Arg1 was significantly decreased, reaching levels similar to or even lower than those in the Veh‐TBI group (Figure 5F). These results indicate that the inhibition of Nrf2/HO‐1 axis activation can negate the influence of H19‐KD on microglia/macrophage polarization in the peri‐injury area after TBI, thereby preventing its regulation of neuroinflammation in the brain. In summary, our behavioral and histological experiments demonstrate that inhibiting the Nrf2/HO‐1 axis partially abolishes the neuroprotective effect of H19‐KD after TBI. This implies that the neuroprotective effect of H19‐KD after TBI is at least partially mediated by the activation of the Nrf2/HO‐1 axis.

## 4 DISCUSSION

Previous studies on H19 have demonstrated its upregulation associated with neurotrauma across various CNS diseases. Inhibiting its expression has exerted neuroprotective effects.^20^, ^21^, ^46^ Similarly, elevated expression levels of H19 have been observed in TBI models, correlating with neurological functional impairments. This phenomenon may be related to the hypoxic conditions present in local CNS injury. The upregulation of H19 is responsive to increased activity of hypoxia‐inducible factor 1α (Hif‐1α)^47^ and is sensitive to hypoxic stimulation. In the MCAO model, levels of H19 positively correlate with the severity of ischemic/hypoxic injury.^42^ Considering the disease progression of TBI, local ischemia and hypoxia in the brain often result from various primary injuries, especially in patients with multiple traumas and unstable systemic circulation. Therefore, this pivotal process in TBI pathology may elucidate the alterations in H19 expression after TBI, offering novel insights for further exploration of CNS injury treatment strategies involving H19.

In clinical practice, moderate to severe TBI often results in irreversible primary neuronal tissue damage. Alongside this primary damage, secondary structural damage occurs in both gray and white matter, characterized by neuronal loss, axonal demyelination, and other abnormalities.^48^, ^49^ These secondary injuries further exacerbate sensory, motor, and cognitive impairments in patients.^9^, ^50^ Our study demonstrates that H19‐KD significantly reduced neuronal loss in the perilesional CTX and STR in the TBI model, indicating its potential as a protective measure against gray matter injury. White matter, comprising half of the brain and crucial for advanced cognitive functions, is particularly susceptible to extensive neuropathological damage compared to gray matter.^51^, ^52^, ^53^, ^54^, ^55^ With the growing recognition of white matter function, research into white matter injury has been identified. Previous studies have shown that lncRNA can affect the repair of axons or myelin sheath after injury by directly participating in or regulating miRNA.^56^, ^57^, ^58^ However, there is limited understanding of how H19 contributes to white matter regulation. Our study is the first to demonstrate, in the context of TBI, that H19‐KD attenuates myelin loss and axonal damage in CTX and STR surrounding the injury site, offering promising protection against white matter injury.

TBI significantly increases the risk of Alzheimer's disease (AD), with Aβ plaques detected in both acute and chronic stages of severe TBI patients. Primary mechanical injuries disrupt axonal structure and function, leading to Aβ deposition. This, in turn, triggers a series of pathophysiological events, exacerbating secondary gray and white matter damage.^44^, ^59^, ^60^ Our study also observed a significant increase in Aβ deposition in the CC region after TBI, while H19‐KD notably reduced Aβ deposition in this area. However, since we did not localize Aβ to the site of axonal injury after TBI, further investigation is required to understand how axonal injury leads to Aβ deposition and whether H19 modulates Aβ deposition by influencing axonal injury.

Microglia, as brain‐resident macrophages, maintain a steady‐state phenotype under normal physiological conditions, continuously monitoring the brain parenchyma. Upon injury, they rapidly activate and migrate to the site of injury, adopting different polarization states according to environmental changes.^61^, ^62^ A growing number of microglial phenotypes have been identified in development, aging, and disease, challenging previous classification methods.^63^, ^64^ This study primarily focuses on describing microglia/macrophage polarization based on their functions in the inflammatory state, categorizing them into pro‐inflammatory and anti‐inflammatory phenotypes. Numerous studies have highlighted the role of H19 in regulating microglia/macrophage activation across different neurological diseases, impacting the inflammatory response.^29^, ^30^, ^65^, ^66^ Our study, for the first time in a TBI mouse model, demonstrated that H19‐KD modulates the inflammatory polarization of microglia/macrophages, thereby alleviating neuroinflammation and laying the foundation for further investigation into H19 in TBI. Additionally, the prognosis of stroke patients has shown positive correlations with the expression levels of H19 and TNF‐α in the blood, suggesting their potential as diagnostic indicators with high sensitivity and specificity.^30^ This implies that assessing H19 expression in peripheral blood and cerebrospinal fluid, combined with clinical indicators, could emerge as a novel method for evaluating TBI clinical status and predicting prognosis.

The neuroinflammatory response, a key secondary injury process in TBI, involves diverse signaling pathways and molecular mechanisms, including Nrf2, forming a complex regulatory network.^31^, ^67^, ^68^ As a transcription factor, Nrf2 regulates the transcription of numerous genes. Under normal conditions, Nrf2 binds to Keap1, residing in the cytoplasm. Upon stress events such as injury, Nrf2 translocates to the nucleus, activating downstream molecules like HO‐1 expression, thus participating in various pathophysiological processes.^69^, ^70^, ^71^ Notably, Nrf2 contributes significantly to the regulation of microglia/macrophage‐mediated inflammatory responses,^35^, ^72^, ^73^, ^74^ exerting neuroprotective effects. Genome‐wide mapping analysis reveals Nrf2 binding sites in the distal genomic regions, possibly including the promoter region of lncRNA,^75^ promoting researchers to explore the regulatory role of lncRNA in the Nrf2/HO‐1 axis. While limited studies have implicated H19 in regulating Nrf2/HO‐1 expression in myocardial injury,^40^, ^41^ its role in CNS diseases like TBI remains unexplored. Here, our study demonstrates that H19‐KD activates the Nrf2/HO‐1 axis, exerting neuroprotective effects and improving neurological function post‐TBI. Given that the AAV used in this experiment causes widespread infection of brain tissue 14 days after injection and lacks cellular specificity,^76^ it is impossible to determine the effect of H19‐KD on a single cell type. Future research could more accurately investigate the specific mechanisms of H19‐KD in different cell types after TBI by using primary cell cultures or cell‐specific knockout mice for experiments. Additionally, further studies could focus on the cellular localization of Nrf2 upregulation following H19 inhibition and employ targeted research approaches to elucidate the molecular mechanisms and cellular processes influencing neuroprotection via Nrf2/HO‐1 axis activation.

In addition, this study's exclusive use of male mice introduces several limitations. Incorporating both male and female mice in experiments presents challenges, such as the increased sample size, which imposes additional resource and ethical burdens. Furthermore, sex‐specific factors may differentially influence experimental outcomes. When considering sex as a variable, standardized statistical methods are essential to ensure the accuracy and reproducibility of results.^77^ Including both male and female mice and reporting experimental variables, including sex, may uncover potential sex‐specific responses to treatments, thereby enhancing the reliability and generalizability of the findings. This is crucial for subsequent translational research.

## 5 CONCLUSION

Our study shows that H19 knockdown in the brain significantly alleviates neuronal damage and promotes the activation of microglia/macrophages toward an anti‐inflammatory phenotype, thereby ameliorating neuroinflammation. The key point is that the activation of the Nrf2/HO‐1 axis induced by H19 knockdown leads to improved neurological recovery after TBI.

## AUTHOR CONTRIBUTIONS

Yanqin Gao Leileil Mao and Gang Wu designed this study. Qiankang Chen, Biwu Wu, Ziyu Shi, Yana Wang, Xingdong Chen, and Yuqing Wang performed experiments. Qiankang Chen and Ziyu Shi analyzed the data. Qiankang Chen, Yiwen Yuan, and Leilei Mao wrote the manuscript. Yanqin Gao and Jin Hu critically edited the manuscript. The authors read and approved the final manuscript.

## FUNDING INFORMATION

This work was supported by STI 2030‐Major Projects (2021ZD0201704, 2022ZD0204704), National Natural Science Foundation of China (82071311), Research Initiation Fund of Huashan Hospital Affiliated to Fudan University (2021QD044), Shanghai Municipal Science and Technology Major Projects (22ZR1413700, 2018SHZDZX01), ZJLab, and Shanghai Center for Brain Science and Brain‐Inspired Technology.

## CONFLICT OF INTEREST STATEMENT

All authors declare that they have no competing interests.

## Supporting information


Data S1.

## Data Availability

The data used to support the findings of this study are available from the corresponding author upon request.
