# Supplementary material for: LncRNA H19 knockdown promotes neuropathologic and functional recovery via the Nrf2/HO‐1 axis after traumatic brain injury
Source: CNS Neurosci Ther. 2024 Jul 25;30(7):e14870. doi: 10.1111/cns.14870 (PMC11269889; doi:10.1111/cns.14870)
Supplement: Supplementary file 1 — Data S1. [file CNS-30-e14870-s001.docx]

**LncRNA H19 knockdown promotes neuropathologic and functional recovery via the Nrf2/HO-1 axis after traumatic brain injury**

Qiankang Chen^#^, Biwu Wu^#^, Ziyu Shi^#^, Yana Wang, Yiwen Yuan, XingdongCheney, Yuqing Wang, Jin Hu, Leilei Mao*, Yanqin Gao*, Gang Wu*

Department of Neurosurgery of Huashan Hospital, State Key Laboratory of Medical Neurobiology and MOE Frontiers Center for Brain Science, Institutes of Brain Science, Fudan University, Shanghai 200032, China

^#^ Qingkang Chen, Biwu Wu, and Ziyu Shi contributed equally to this work.

Address Correspondence to:

Dr. Yanqin Gao ([yqgao@shmu.edu.cn](mailto:yqgao@shmu.edu.cn)), Dr. Leilei Mao ([llmao@fudan.edu.cn](mailto:llmao@fudan.edu.cn)), or Gang Wu (wugang3@huashan.org.cn)

Department of Neurosurgery of Huashan Hospital, State Key Laboratory of Medical Neurobiology and MOE Frontiers Center for Brain Science, Institutes of Brain Science, Fudan University, Shanghai 200032, China

**Supplementary materials**

**Supplementary Figures**


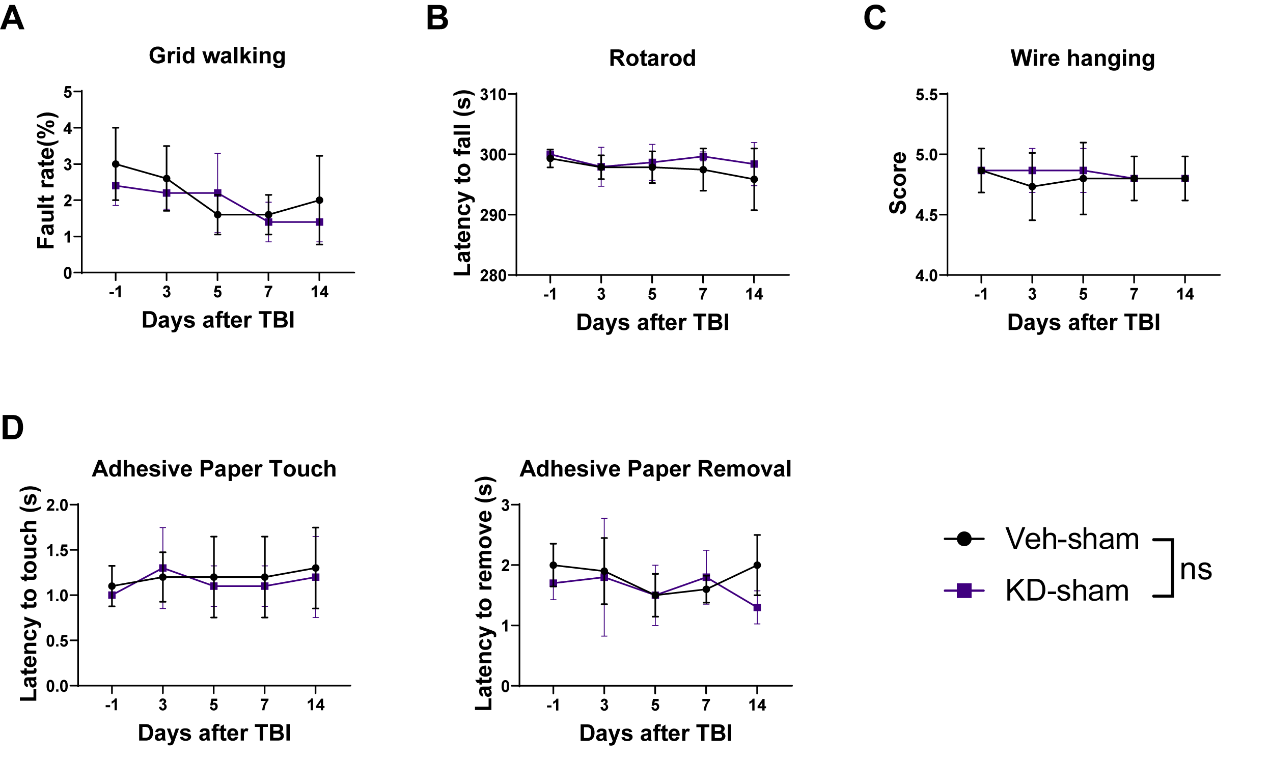


**Figure S1. H19-KD did not affect sensorimotor function under normal physiological conditions.** (A) Foot fault rate in the grid walking test. (B) Latency to fall in the rotarod test. (C) Relative score in the wire hanging test. (D) Latency to touch and remove the tape in the adhesive paper test (n=5/group). Data are presented as mean ± SD. Statistical analyses were performed by Unpaired t-test or one/two-way ANOVA and Tukey's multiple comparisons test. ns: no significance, as indicated.


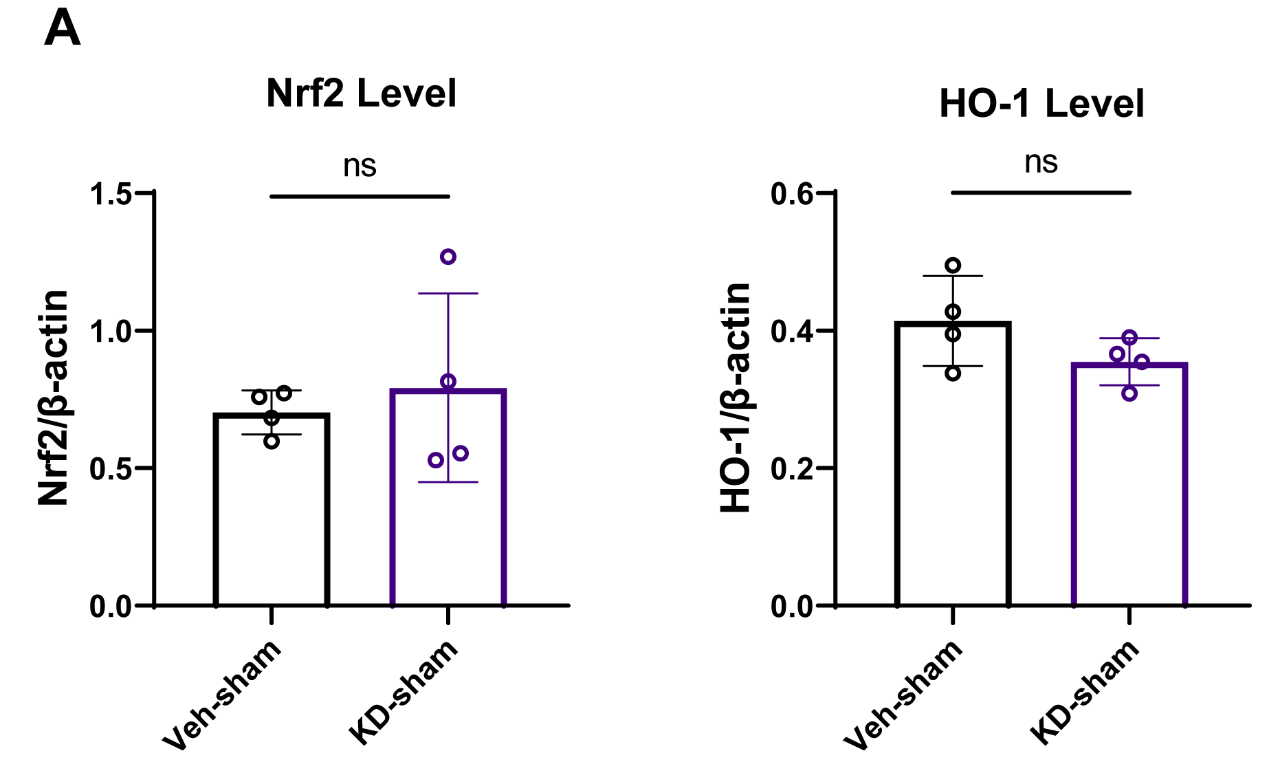


**Figure S2. H19-KD did not affect Nrf2 and HO-1 expression under normal physiological conditions in mice.** (A) Quantitative analysis of Nrf2 and HO-1 protein levels in the sham group of Veh and KD mice (n=4/group). Data are presented as mean ± SD. Statistical analyses were performed by Unpaired t-test with two tails. ns: no significance, as indicated.


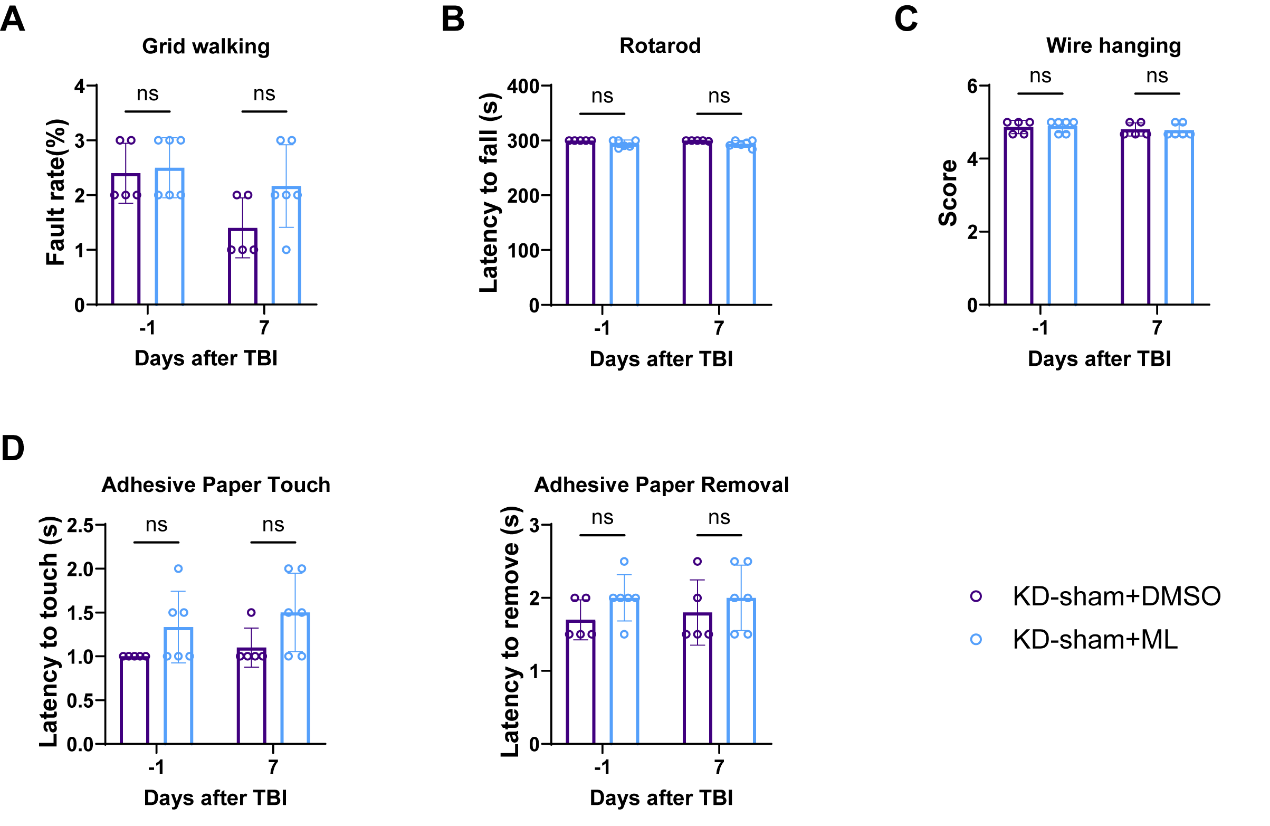


**Figure S3. ML385 injection did not lead to a worse outcome in mice in normal physiological states.** (A) Foot fault rate in the grid walking test. (B) Latency to fall in the rotarod test. (C) Relative score in the wire hanging test. (D) Latency to touch and remove the tape in the adhesive paper test (n=5-6/group). Data are presented as mean ± SD. Statistical analyses were performed by Unpaired t-test or one/two-way ANOVA and Tukey's multiple comparisons test. ns: no significance, as indicated.

**Methods:**

**AAV injection**

The vector, named GV478, contains the component sequence U6-MCS-CAG-EGFP and belongs to serotype 9. The virus titer is 1.06×1013 vg/mL. The interference target sequence of the AAV-shRNA H19 virus is 5'-GGTCCTGGGTCCATCAATAAA-3'; the insert sequence for the control group is 5'-CGCTGAGTACTTCGAAATGTC-3'.

Mice were induced with 3% isoflurane anesthesia in a gas anesthesia chamber and then fixed on the stereotaxic apparatus (Model 900, Kopf Instruments). Anesthesia and respiration were maintained with 1.5% isoflurane using a ventilator, and body temperature was maintained with a warming lamp. After disinfection and removal of hair, a midline incision was made on the scalp to expose the skull. A hole was drilled 0.1 mm posterior to the bregma and 1.0 mm lateral to the midline for injection. A total of 4 μL of AAV or negative control virus solution was injected using a microinjection pump at a rate of 0.4 μL/min to a depth of 2.5 mm. After injection, the needle was left in place for 10 min before slowly withdrawing, and the scalp was carefully sutured. Mice were then placed on a heating pad to maintain body temperature until fully recovery from anesthesia.

**Neurofunctional behavioral tests**

The neurobehavioral deficits and recovery following TBI were evaluated using a range of behavioral experiments. Before TBI surgery, mice underwent 3 days of sensory-motor pre-training to establish stable baselines. Sensory motor function was assessed at 3, 5, 7, and 14 days post-surgery, while cognitive memory function was evaluated on day 14.

**The grid-walking test**, also known as the foot fault test, was used to assess the coordination of limb sensory and motor function in mice. Mice were placed on a steel frame (40 cm (L) × 20 cm (W) × 30 cm (H)) consisting of 2 cm × 2 cm square grids. During the pre-training phase, mice were given 10 minutes to acclimate to the frame. On the day before surgery and at corresponding time points post-surgery, mice were placed on the steel frame to freely move, and their movements were recorded via video for 3 min. The total number of steps and the number of foot faults were counted. The foot fault rate = the number of foot faults / the total number of steps × 100%.

**The rotarod test** was used to evaluate the motor balance and endurance of mice. Mice were placed on a rotarod apparatus (Model 47650, Ugo Basile), with the rotarod speed gradually increasing from 5 rpm to 40 rpm within 300 s. The latency to fall from the rotarod was recorded. On the day before surgery and at corresponding time points post-surgery, mice underwent 3 trials per day, with a 15-minute interval between each trial. The data was exhibited as the average latencies from the 3 trials.

**The adhesive removal test** was used to assess the sensory and fine motor abilities of mice. A 3 mm × 4 mm adhesive tape was placed on the center of the injured side forelimb of the mouse and quickly returned to the test cage. The duration it took for the mouse to perceive and remove the adhesive tape was recorded. If the mouse failed to perceive or remove the adhesive tape within 120 seconds, a maximum time of 120 seconds was recorded. On the day before surgery and at corresponding time points post-surgery, mice underwent 3 trials per day, with a 5 min interval between each trial. The data was exhibited as the average time from the 3 trials.

**The wire-hanging test** was used to assess the muscle strength of mice. A 50 cm long steel wire with a diameter of 2 mm was fixed between two 4 cm × 4 cm platforms, positioned 40 cm above the ground on both sides. Mice were suspended by their forelimbs in the center of the wire, and their performance was observed for 1 minute and scored accordingly. The scoring criteria were as follows: 0 indicated the mouse was unable to grasp the wire and fell off; 1 indicated the mouse hung onto the wire with forelimbs but remained motionless; 2 indicated the mouse hung onto the wire with forelimbs and attempted to move toward the platforms; 3 indicated the mouse hung on the wire with forelimbs and one hind limb and moved toward the platforms; 4 indicated the mouse hung on the wire with all four limbs and tail and moved a certain distance toward both sides; 5 indicated the mouse rapidly reached the platforms within 1 min. During the pre-training phase, mice were trained to climb onto the platforms within 1 minute. After surgery, mice underwent 3 parallel experiments per day at corresponding time points post-surgery, with a 15-minute interval between each trial. The comprehensive score was determined by averaging the results of the three trials

**The novel object recognition test** was used to evaluate the recognition and memory function of mice. Fourteen days post-surgery, mice were first placed in an open box (40 cm × 40 cm × 40 cm) and allowed to explore for 10 min to acclimate to the environment. Then, two objects with identical shapes, textures, and colors were placed diagonally across from each other in the box, and the mice were allowed to freely explore for 10 min while being video-recorded. Four hours later, one of the objects was replaced with a new object different from the previous one, and the mice were again allowed to explore freely for 10 min while being video-recorded. Behavioral analysis software (ANY-maze, Global Biotech) was used to analyze the time spent exploring each object. The investigation time proportion = Time novel / (Time novel + Time familiar) × 100%; the discrimination index = (Time novel - Time familiar) / (Time novel + Time familiar) × 100%.

**The three-chamber social test** was utilized to evaluate the social preference and memory function of mice. Mice were placed in a rectangular box (60 cm (L) × 40 cm (W) × 40 cm (H)), divided into three equally sized compartments by partitions. Small doors that could be opened were installed between the adjacent compartments, and an empty cage was placed against the wall in each of the two end compartments. During the adaptation phase, the experimental mice were placed in the central compartment, and the doors on both sides were opened to allow free exploration for 5 min. During the social interaction test phase, a stranger mouse (Stranger 1) was placed in one of the cages on one side, and then the experimental mouse was placed in the central compartment. The door was opened, allowing the experimental mouse to freely explore for 10 min while being video recorded. In the subsequent social novelty preference test phase, another stranger mouse (Stranger 2) was placed in the cage on the other side. The experimental mouse was again allowed to freely explore the three compartments for 10 min while being video recorded. The time spent exploring each side in the two test phases was measured using analysis software. The social preference index was calculated to evaluate the social ability and the social novelty of mice. Between each cognitive behavioral test, the interior of the box was cleaned with 75% ethanol.

**Immunofluorescence staining**

Mice were deeply anesthetized using 1% pentobarbital sodium at a dosage of 50 ml/kg. Then the chest was incised to expose the heart, and 4°C PBS and 4% paraformaldehyde (PFA) were perfused into the heart at a constant rate. The intact brain was then carefully extracted and placed in 4% PFA overnight at 4°C, followed by sequential immersion in 20% and 30% sucrose solutions for dehydration. Coronal sections with a thickness of 25 μm were obtained using a cryostat (CM1950, Leica) and stored at -20°C for later use.

The brain sections obtained were rinsed in PBS three times for 5 min each, followed by a 20-minute incubation in 1% PBST for permeabilization, and another rinse in PBS. The sections were then blocked with Blocking Buffer for Immunol Staining (P0260, Beyotime Biotechnology) at room temperature for 20 min. If the primary antibody used was of mouse host, the M.O.M. kit (BMK-2202, Vector Laboratories) was simultaneously used for blocking. Subsequently, the brain sections were incubated overnight at 4°C in the primary antibody working solution. The details of the primary antibodies used in this study and their corresponding dilutions are listed in Table S1. After rinsing in PBS, the sections were incubated with secondary antibodies conjugated with Alexa Fluor-488/594/647 (1:1000, Jackson ImmunoResearch Laboratories) at room temperature for 2 hours. The brain sections were mounted on slides, and DAPI Fluoromount-G (0100-20, Southern Biotech) was added before covering with coverslips. Fluorescence images were captured using an Olympus FV3000 Confocal Laser Scanning Microscope and a Nikon ECLIPSE Ni-E microscope. Image processing and analysis were performed using ImageJ software (Fiji, NIH).

**Cresyl violet Nissl staining**

After rinsing the brain sections with PBS, mount them on slides and dry them in a 37°C oven for 24 hours. Submerge the slides in xylene for 10 min, followed by sequential immersion in 100%, 95%, and 75% ethanol, and finally in ddH_2_O for 5 min each. Subsequently, immerse the slides in 1% cresyl violet staining solution for 2 min, then rinse them thoroughly with ddH_2_O to remove excess dye. Finally, sequentially immerse the slides in 75%, 95%, and 100% ethanol for 2 min each for dehydration, and soak them in xylene for 5 min before sealing them with neutral resin. Capture images using the Olympus VS200 Slide Scanner. Measure and calculate tissue loss using ImageJ: area of tissue loss % = (area of contralateral hemisphere - area of the ipsilateral hemisphere) / area of the contralateral hemisphere; total tissue loss % = ∑(area of contralateral hemisphere - area of the ipsilateral hemisphere) / ∑(area of the contralateral hemisphere) × 100%.

**Real-time qPCR**

After perfusion of mice with 4°C PBS, cortical and striatal tissues within 2 mm of the brain injury were harvested. Total RNA was extracted using the MolPure Tissue Total RNA Kit (19221ES, Yeasen), followed by reverse transcription to obtain cDNA using the RevertAid First Strand cDNA Synthesis Kit (K1622, Thermo Scientific). The reaction system was prepared with SYBR Green Master Mix (11201ES, Yeasen), and RT-qPCR was performed on QuantStudio 5 (A28140, Thermo Science). All reactions were performed in triplicate, and mRNA relative expression levels were analyzed with normalization to GAPDH levels. The primer sequences used are provided in Table S2.

**Western blotting**

Tissue located within 2 mm of the mouse brain injury was harvested and homogenized in RIPA buffer (9806S, Cell Signaling Technology) supplemented with 1 mM PMSF, PhosSTOP phosphatase inhibitor (4906845001, Roche), and cOmplete protease inhibitor (4693132001, Roche). Total protein was extracted through ultrasonic homogenization. Equal amounts of proteins were loaded onto SDS-PAGE gels and subsequently transferred to PVDF membranes (IPVH00010, Millipore). The membranes were initially blocked with 5% BSA for 1 hour, followed by overnight incubation with primary antibodies (Table S1) at 4°C. After rinsing with TBST, the membranes were incubated with the corresponding HRP-conjugated secondary antibodies (1:5000, Cell Signaling Technology) for 1 hour at room temperature. Protein bands were scanned using the Bio-Rad ChemiDoc system, and quantitative analysis was performed using ImageJ software.

Table S1. List of primary antibodies

| Antibody | Host | Dilution | Company | Category |
| --- | --- | --- | --- | --- |
| NeuN | Rabbit | 1:1000 | abcam | ab177487 |
| MBP | Rat | 1:1000 | Sigma-Aldrich | ab7349 |
| NF200 | Rabbit | 1:1000 | abcam | ab8135 |
| Aβ | Mouse | 1:200 | Invitrogen | MA1-25493 |
| Iba1 | Rabbit | 1:1000 | WaKo | KA1019-19741 |
| CD16 | Mouse | 1:200 | BD Pharmingen | 553142 |
| Arg1 | Rat | 1:100 | Santa Cruz | SC-271430 |
| Nrf2 | Rabbit | 1:500(IF)  1:1000(WB) | Proteintech | 16396-1-AP |
| HO-1 | Rabbit | 1:1000(WB) | Proteintech | 10701-1-AP |
| β-actin | Mouse | 1:5000(WB) | Yeasen | 30101ES |

Table S2. List of RT-qPCR primer

| Gene name | Primer sequences |
| --- | --- |
| LncRNA H19 | Forward：5'-TCCCAGAACCCACAACATGAA-3'  Reverse：5'-TTCACCTTCCAGAGCCGATTC-3' |
| CD16 | Forward：5'-TTTGGACACCCAGATGTTTCAG-3'  Reverse：5'-GTCTTC CTTGAGCACCTGGATC-3' |
| CD32 | Forward：5'-AATCCTGCCGTTCCTACTGATC-3'  Reverse：5'-GTGTCACCGTGTCTTC CTTGAG-3' |
| CD11b | Forward：5'-CCAAGACGATCTCAGCATCA-3'  Reverse：5'-TTCTGGCTTGCTGAATCCTT-3' |
| CD86 | Forward：5'-GACCGTTGTGTGTGTTCTGG-3'  Reverse：5'-GATGAGCAGCATCACAAGGA-3' |
| IL-1β | Forward：5'-CTCCATGAGCTTTGTACAAGG-3'  Reverse：5'-TGCTGATGTACCAGTTGGGG-3' |
| IL-6 | Forward：5'-ACACATGTTCTCTGGGAAATC-3'  Reverse：5'-AGTGCATCATCGTTGTTCATA-3' |
| TNF-α | Forward：5'-GACCCTCACACTCAGATCATCTTCT-3'  Reverse：5'-CCTCCACTTGGTGGTTTGCT-3' |
| iNOS | Forward：5'-CAAGCACCTTGGAAGAGGAG-3'  Reverse：5'-AAGGCCAAACACAGCATACC-3' |
| CD206 | Forward：5'-CAAGGAAGGTTGGCATTTGT-3'  Reverse：5'-CCTTTCAGTCCTTTGCAAGC-3' |
| IL-10 | Forward：5'-GCTGTCATCGATTTCTCCCCT-3'  Reverse：5'-GACACCTTGGTCTTGGAGCTTAT-3' |
| Arg1 | Forward：5'-CTCCAAGCCAAAGTCCTTAGAG-3'  Reverse：5'-AGGAGCTGTCATTAGGGACATC-3' |
| TGF-β | Forward：5'-TGCGCTTGCAGAGATTAAAA-3'  Reverse：5'-CGTCAA AAGACAGCCACTCA-3' |
| GAPDH | Forward：5'-GTGAAGGTCGGTGTGAACGG-3'  Reverse：5'-GTTTCCCGTTGATGACCAG-3' |
